# The SARS-CoV-2 spike residues 616/644 and 1138/1169 delineate two antibody epitopes in COVID-19 mRNA COMIRNATY vaccine (Pfizer/BioNTech)

**DOI:** 10.1038/s41598-022-10057-7

**Published:** 2022-04-09

**Authors:** Jessica Andries, Wildriss Viranaicken, Colette Cordonin, Charline Herrscher, Cynthia Planesse, Bénédicte Roquebert, Marie Lagrange-Xelot, Chaker El-Kalamouni, Olivier Meilhac, Patrick Mavingui, David Couret, Gilles Gadea, Philippe Despres

**Affiliations:** 1 Université de La Réunion, INSERM U1187, CNRS UMR 9192, IRD UMR 249, Unité Mixte Processus Infectieux en Milieu Insulaire Tropical (PIMIT), Plateforme Technologique CYROI, 94791 Sainte Clotilde, La Réunion, France; 2 Plate-Forme Technologique CYROI, 94791 Sainte Clotilde, La Réunion, France; 3 Université de La Réunion, INSERM U1188, Unité Mixte Diabète Athérothrombose Thérapies Réunion Océan Indien (DeTROI), Plateforme Technologique CYROI, 94791 Sainte Clotilde, La Réunion, France; 4 Laboratoire CERBA, Parc d’activités « Les Béthunes », 95310 Saint-Ouen-l’Aumône, France; 5 Centre Hospitalier Felix Guyon, Centre Hospitalier Universitaire-La Réunion, 97000 Saint-Denis, La Réunion, France; 6 Groupe Hospitalier Sud Réunion, Centre Hospitalier Universitaire-La Réunion, 97410 Saint-Pierre, La Réunion, France; 7 Present Address: IRCM, U1194, MetaSarc Team, 34298 Montpellier, France

**Keywords:** Immunology, Microbiology

## Abstract

The newly identified coronavirus SARS-CoV-2 is responsible for the worldwide pandemic COVID-19. Considerable efforts have been devoted for the development of effective vaccine strategies against COVID-19. The SARS-CoV-2 spike protein has been identified as the major antigen candidate for the development of COVID-19 vaccines. The Pfizer-BioNTech COVID-19 vaccine comirnaty is a lipid nanoparticle-encapsulated mRNA encoding a full-length and prefusion-stabilized SARS-CoV-2 spike protein. In the present study, synthetic peptide-based ELISA assays were performed to identify linear B-cell epitopes into the spike protein that contribute to elicitation of antibody response in comirnaty-vaccinated individuals. The synthetic S2P6 peptide containing the spike residues 1138/1169 and to a lesser extent, the synthetic S1P4 peptide containing the spike residues 616/644 were recognized by the immune sera from comirnaty vaccine recipients but not COVID-19 recovered patients. We assume that the synthetic S2P6 peptide and to a lesser extent the synthetic S1P4 peptide, could be of interest to measure the dynamic of antibody response to COVID-19 mRNA vaccines. The S2P6 peptide has been identified as immunogenic in adult BALB/c mice that received protein-peptide conjugates in a prime-boost schedule. This raises the question on the role of the B-cell epitope peptide containing the SARS-CoV-2 spike residues 1138/1169 in protective efficacy of the Pfizer-BioNTech COVID-19 vaccine comirnaty.

## Introduction

SARS-CoV-2 virus is an enveloped RNA virus belonging to betacoronavirus genus of *Coronoviridae* family^1^. The COVID-19 disease associated with the emerging SARS-CoV-2 infection is a pandemic public health threat since early 2020 with millions of deaths to date^1^. The main route for SARS-CoV-2 transmission is through respiratory droplets necessitating the implementation of effective control measures^2–6^. Convalescent COVID-19 patients develop neutralizing anti-SARS-CoV-2 antibodies produced by the antiviral immune response^7–9^. The structural spike S protein playing a crucial role in eliciting the immune response during COVID-19 disease has been considered as the predominant viral antigen target for vaccine development^10–13^. The transmembrane homo-trimer spike protein at the virus surface mediates receptor binding through interaction with cell entry receptor ACE2^14–18^. The spike protein is composed of the S1 and S2 subunits^10,11,13^. The N-terminal S1 subunit which comprises the Receptor Binding Domain (RBD) triggers binding to ACE2 receptor and anti-RBD antibodies were shown to exert potent neutralizing activity against SARS-CoV-2^10–12^. The C-terminal S2 subunit containing the fusion elements driving viral and host-cell membrane fusion^10–12^. A polybasic insertion RRAR that can be cleaved by furin has been identified at the S1/S2 boundary playing a major role in the pathogenesis of SARS-CoV-2 infection^14–17^. The virus binding to ACE2 receptor via the RBD elicits the proteolytic cleavage of pre-protein S into S1 and S2 subunits depending of cell surface protease TMPRSS2^18–20^. Such processing results in large conformational changes causing S1 shedding and fusion elements exposure in S2 leads to a fusion process between viral and host-cell membranes in an endosome-independent pathway^10,14,21,22^.

Accelerated vaccine programs for COVID-19 prevention have led to the development of commercially available vaccines targeting the SARS-CoV-2 spike protein^13,23–25^. To date, the most performing current vaccines are based on nucleoside-modified mRNA strategy^26–29^. The COVID-19 mRNA BNT162b2 vaccine which has been known as the Pfizer-BioNTech COVID-19 vaccine (brand name comirnaty) is a lipid nanoparticle-encapsulated mRNA encoding a full-length and prefusion-stabilized S protein^13,26–29^. The comirnaty vaccine was initially administered in two doses, 21 days apart, and now in three and even four doses. The approval of this candidate vaccine has been based on a large group of individuals who had been immunized with BNT162b2 in an international and placebo-controlled phase III trial^28,29^. Vaccinated individuals developed high neutralizing SARS-CoV-2 antibody titers which are indicators of COVID-19 protection^28,29^. The efficacy study demonstrated that COVID-19 mRNA BNT162b2 vaccine candidate induced 95% protection against COVID-19 in a 2-dose regimen^28,29^.

To our knowledge, there has been little information on specificities of antibodies induced by comirnaty vaccine. With the aim to design peptide-based vaccine candidates for COVID-19 prevention, we were interested in identifying linear antibody epitopes which are potentially targets of anti-spike antibodies in individuals who have been immunized with COVID-19 mRNA comirnaty vaccine. Neutralization linear antibody epitopes for SARS-CoV-2 have been identified in COVID-19 patients^30–33^. In the present study, we examined the antibody response post-vaccination with the comirnaty vaccine. Synthetic peptides representing potential B-cell epitopes in the SARS-CoV-2 spike protein were assessed for their antigenic reactivity with immune serum from comirnaty-vaccinated individuals. We showed that administration of comirnaty vaccine to an infection-naïve individual or COVID-19 immune subject elicits a production of antibodies capable of binding to two linear B-cell epitope peptides which are identified in the S1 and S2 subunits of the spike protein.

## Results

### Synthetic peptides representing potential linear B-cell epitopes in the spike protein

To search for possible linear B-cell epitopes in SARS-CoV-2 spike protein, we used the predictor tool BepiPred-2.0 available in IEDB (for The Immune Epitope Database and Analysis Resource) website. We submitted the spike protein sequence from Wuhan strain of SARS-CoV-2 using 0.50 as cutoff parameter and 23 potential epitope peptides were identified (Table S1). Considering that long peptides would be more appropriate to retain conformational information related to efficiency of antibody binding, we selected a limited number of candidate B-cell epitope peptides around 20 to 30 residues spanning the S1 and S2 subunits of SARS-CoV-2 spike protein (Table 1). Consequently, only the B-cell epitope peptides # 1, 9, 10, 13, 14, 16, and 22 were designed to generate the synthetic peptides S1P1, S1P2, S1P3, S1P4, S1P5 and S2P6 (Table 1). The 29-mer synthetic peptide S1P4 results of the combination of the two adjacent B-cell epitope peptides #13 and #14 whereas 32-mer synthetic peptide S2P6 peptide is based on B-cell epitope peptide # 22.

**Table 1:** Sequences of synthetic peptides and their variants.

Peptide	Amino acid sequence	Spike residues	Length
S1P1	SQCVNLTTRTQLPPAYTNSFTRGVY	13–37	25-mer
S1P1^[F,N,s]^*	SQCVNFTNRTQLPSAYTNSFTRGVY	13–37	25-mer
S1P2	YNSASFSTFKCYGVSPTKLNDLCF	369–392	24-mer
S1P3	GDEVRQIAPGQTGKIADYNYKL	404–425	22-mer
S1P3^[N]^**	GDEVRQIAPGQTGNIADYNYKL	404–425	22-mer
S1P4	NCTEVPVAIHADQLTPTWRVYSTGSNVFQ	616–644	29-mer
S1P5	ASYQTQTNSPRRARSVASQ	672–690	19-mer
S1P5^[H]^***	ASYQTQTNSHRRARSVASQ	672–690	19-mer
S2P6	YDPLQPELDSFKEELDKYFKNHTSPDVDLGDI	1138–1169	32-mer
S2P6.2.0	SFKEELDKYFKNHTSPDVD	1147–1165	19-mer

*Gamma, **Beta/Omicron, and ***Alpha variants of SARS-CoV-2 according to WHO label. The amino-acid substitutions are underlined.

**Figure 1:**
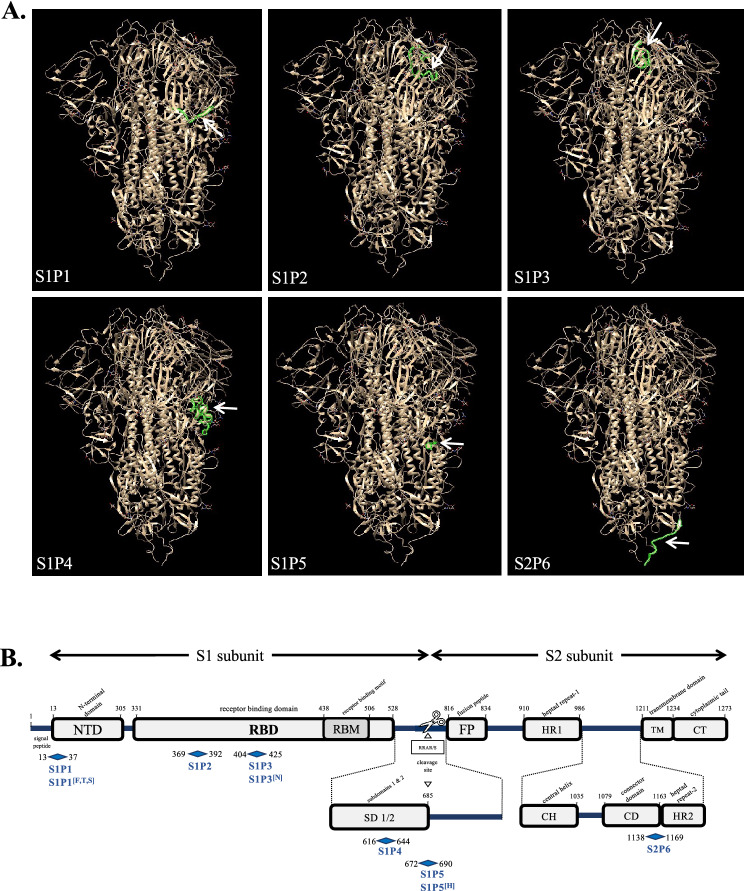
Position of B-cell epitope peptides on SARS-CoV-2 spike protein. In (**A**), positions of the six B-cell epitope peptides colored in green on a single protomer of SARS-CoV-2 spike protein trimer (EMD data resource: EMD-2256). The blank arrow indicates their position on the protomer. In (**B**), schematic organization of SARS-CoV-2 spike protein with the S1 and S2 subunits and their different domains and motifs is shown. The six synthetic peptides S1P1, S1P2, S2P3, S1P4, S1P5, and S2P6 peptides and their variants S1P1^[F,T,S]^, S1P3^[N]^, and S1P5^[H]^ are mapped on the SARS-CoV-2 spike protein.

The six identified linear B-cell epitope peptides were mapped on the tridimensional structure prediction of SARS-CoV-2 spike protein trimer (Fig. 1A). The B-cell epitope peptides S1P4 and S2P6 were exposed at the surface of the protomer whereas S1P1, S1P2, S1P3, S1P4 and S1P5 were orientated inside of the structure. In the SARS-CoV-2 spike protein, the S1P1, S1P2, S1P3, S1P4, and S1P5 peptides are identified in the N-terminal domain (NTD), receptor binding domain (RBD), and C-terminal domain 2 (CTD2) sequences of the S1 subunit, respectively (Fig. 1B). The S2P6 peptide spans the connector domain (CD) and heptad repeat-2 (HR2) sequences into the C-terminal region of the S2 subunit (Fig. 1B). To note that the B-cell epitope peptide S2P6 which contains a putative N-glycosylation motif ^1158^NHT^1160^ is strictly conserved between SARS-CoV-2 and SARS-CoV spike proteins^21^.

The Alpha, Beta, Gamma, Delta, and now Omicron variants of SARS-CoV-2 have been classified as dominant VOC (Variants of Concern) by World Health Organization. Given that SARS-CoV-2 variants display an increased transmissibility and/or change on viral pathogenicity, we developed S1P1^[F,N,S]^, S1P3^[N]^, and S1P5^[H]^ variant peptides bearing amino-acid substitutions that have been identified in VOC (Table 1). We noted that amino-acid changes identified in the spike protein of recent VOC including Delta and Omicron variants have no impact on both S1P4 and S2P6 peptides.

### Antigenic reactivity of synthetic peptides with comirnaty vaccine recipient sera

**Figure 2:**
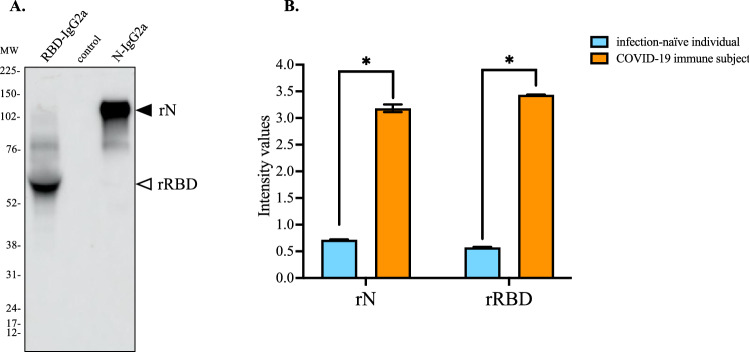
Antigenic reactivity of recombinant N and RBD proteins with human serum-specific SARS-CoV-2 antibody. In (**A**), CHO cells were transfected 72 h with plasmids expressing RBD-IgG2A (rN) and N-IgG2A (rRBD) proteins or empty vector plasmid (control). Aliquots of clarified cell culture supernatants (C3S) were analyzed by immunoblot assay using goat anti-mouse IgG heavy chain HRP antibody. The original full-length gel is presented in Supplementary Figure S1. In (**B**), pools of four COVID-19 donor serum samples (Table S2, serum donors n°16, 18, 19, and 20) (COVID-19 donor serum) and four negative donor serum samples (Table S3) (negative donor serum) at dilution 1:100 were tested for anti-N and anti-rRBD antibodies by indirect ELISA using rN and rRBD for protein-based antibody capture. The intensity values of serum samples were measured at O.D. 450 nm. The results are the mean (± SEM) of three replicates. Unpaired *t* tests between COVID-19 immune subject and infection-naïve individual were performed and statistically significant comparisons are shown as * *p* < 10^–4^.

To evaluate the antibody response against SARS-CoV-2 spike protein in comirnaty-vaccinated individuals, we developed an indirect ELISA assay based on soluble recombinant RBD protein (rRBD) produced from transfected CHO cells. A recombinant soluble SARS-CoV-2 nucleoprotein N protein (rN) was also produced as control antigen for natural infection. Both both rN and rRBD were preceded by a heterologous signal peptide and ended by the IgG2A heavy chain region as C-terminal protein tag. The secreted rN and rRBD proteins from transient transfected CHO cells were detected by immunoblot assay (Fig. 2A). Soluble rN and rRBD proteins were quantified by direct ELISA assay using goat anti-mouse IgG heavy chain HRP antibody. Serum samples from COVID-19 immune subjects (Table S2) and infection-naïve individuals (Table S3) were used to validate the antigenic reactivity of rN and rRBD proteins by indirect ELISA (Fig. 2B).

**Figure 3:**
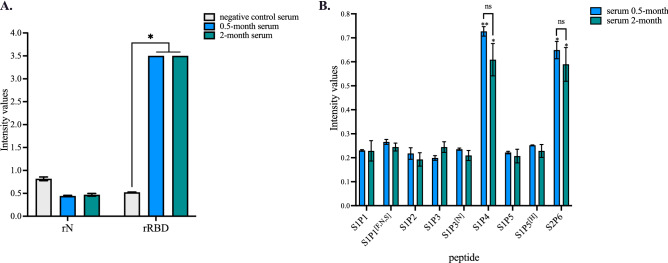
Antigenic reactivity of synthetic peptides with comirnaty vaccine recipient sera. Serum samples from an infection-naïve individual who received comirnaty vaccine in a 2-dose regimen were collected at 0.5 month or 2 months after the injection of a second dose. A pool of ten infection-naïve individuals collected in 2019 (Table S3) served as negative control serum. In (**A**), serum samples at dilution 1:100 were assessed for the detection of antibodies against SARS-CoV-2 N and S proteins by indirect ELISA using soluble rN and rRBD proteins for antigen-based antibody capture. The intensity values of serum samples were measured at O.D. 450 nm. The results are the mean (± SEM) of three replicates. Statistical comparisons were performed between serum samples. Statistically significant comparisons are shown as * *p* < 10^–4^ (ns: non-statistically significant, *p* > 0.05). In (**B**), the serum samples were assayed at dilution 1:50 on synthetic peptides and their variants (200 ng.ml^−1^) through synthetic peptide-based ELISA. The intensity values of vaccine serum sample were measured at O.D. 450 nm. The results are the mean (± SEM) of three replicates. Pairwise comparisons between peptides showed that the experimental points for S1P4 and S2P6 peptides are significantly different from the other peptides (** *p* < 10^–4^, * *p* < 10^–3^). The differences between 0.5 month and 2.0 month for the synthetic S1P4 and S2P6 peptides are non-statistically significant (ns, *p* > 0.05).

The antigenic reactivity of synthetic peptides was evaluated with the serum sample from an infection-naïve individual who has been fully immunized with comirnaty vaccine in April 2021. The immune reactivity of comirnaty vaccine recipient serum was first evaluated on SARS-CoV-2 spike protein by indirect ELISA using rRBD protein for spike protein-based antibody capture (Fig. 3A). A pool of serum samples from ten infection-naïve subjects (Table S3) served as negative serum control. Administration of comirnaty vaccine resulted in significant production of anti-RBD antibodies in vaccinated subject (Fig. 3A). A high anti-RBD antibody titer was maintained for 2 months after injection of a second vaccine dose (Fig. 3A). As a measure of immunity to natural infection, a lack of antibody response against rN confirmed that individual remained negative for SARS-CoV-2 infection at the time of vaccination (Fig. 3A).

**Figure 4:**
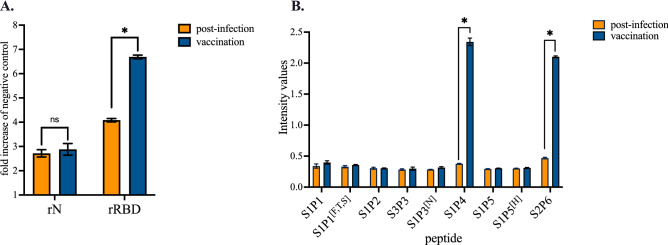
Antigenic reactivity of synthetic peptides with immune sera from a COVID-19 immune subject who received comirnaty vaccine. Serum samples from a COVID-19 patient were collected few weeks after recovery of SARS-CoV-2 infection (post-infection) and then two weeks after the injection of a single dose of comirnaty vaccine (vaccination). Vaccine administration was performed three months after COVID-19 recovery. In (**A**), serum samples at dilution 1:100 were assayed for the detection of antibodies against SARS-CoV-2 N and spike proteins by indirect ELISA using recombinant rN and rRBD proteins for antigen-based antibody capture. A pool of serum samples from infection-naïve individuals (Table S3) served as negative control serum. The intensity values of serum samples were measured at O.D. 450 nm and their immune reactivity was estimated as a fold increase of intensity values obtained with negative control serum. The results are the mean (± SEM) of three replicates. Statistically significant comparisons are shown as * *p* < 10^–4^ (ns: non-statistically significant, *p* > 0.05). In (**B**), serum samples at dilution 1:50 were assayed for the detection of specific antibodies through peptide-based ELISA. The intensity values of serum samples were measured at O.D. 450 nm. The results are the mean (± SEM) of three replicates. Paired *t* tests on experimental points between post-infection and vaccination immune were performed and statistically significant comparisons are shown as * *p* < 10^–4^. Differences between experimental points considered as non-statistically significant are not shown.

The immune serum from comirnaty-vaccinated individual was assayed for reactivity of antibodies against the synthetic peptides through a peptide-based ELISA (Fig. 3B). Among the six synthetic peptides and their variants, only S1P4 and S2P6 were significantly recognized by the immune serum from the vaccinated subject at serum dilution 1:50. As such, this result is consistent with the accessibility of the two B-cell epitope peptides at the surface of the SARS-CoV-2 spike protein trimer (Fig. 1A). The immune reactivity of comirnaty vaccine recipient serum against the synthetic S1P4 and S2P6 peptides was observed up to two months after the injection of a second vaccine dose (Fig. 3B). We then wondered whether administration of comirnaty vaccine to an individual who had experienced SARS-CoV-2 infection induced production of antibodies targeting the synthetic peptides. Indeed, it has been reported that a single dose of a such COVID-19 mRNA vaccine is sufficient to maximize immune protection in post-infection individuals^34^. In line with this finding, antibody capture assays were performed on a serum sample from a COVID-19 immune subject who had received a single-dose of comirnaty vaccine three months after recovery from mild disease. The diagnosis of SARS-CoV-2 infection had been performed by PCR during the acute phase of COVID-19. Administration a single dose of vaccine significantly increased the level of anti-RBD antibodies but not anti-N antibodies in COVID-19 immune subject (Fig. 4A). Thus, comirnaty vaccine elicits antibody response against the SARS-CoV-2 spike protein in a COVID-19 immune subject. The reactivity of COVID-19 patient serum was assayed few weeks after recovery through synthetic peptide-based ELISA (Fig. 4B). We observed a lack of antigenic reactivity for all the synthetic peptides tested in relation to COVID-19 donor serum. Thus, the stretches of spike residues 616/644 and 1138/1169 were not or weakly immunogenic in an individual who had experienced a natural infection with SARS-CoV-2. Administration of comirnaty vaccine in COVID-19 recovered individual resulted in production of antibody targeting synthetic S1P4 and S2P6 peptides 0.5 month after injection of a single dose (Fig. 4B). Thus, comirnaty vaccine was efficient to elicit antibody response against the stretches of spike residues 616/644 and 1138/1169 in a COVID-19 patient who previously mounted a humoral immunity against SAR-CoV-2 spike protein in the course of a natural infection. The levels of antibodies directed against the synthetic S1P4 and S2P6 peptides were higher in the vaccinated individual who was previously infected with SARS-CoV-2 than those without prior evidence infection. This is consistent with the finding that vaccination increased anti-SARS-CoV-2 spike antibodies in COVID-19 immune subjects in a greater magnitude than after a prime-boost vaccine strategy in infection-naïve individuals^35^.

**Figure 5:**
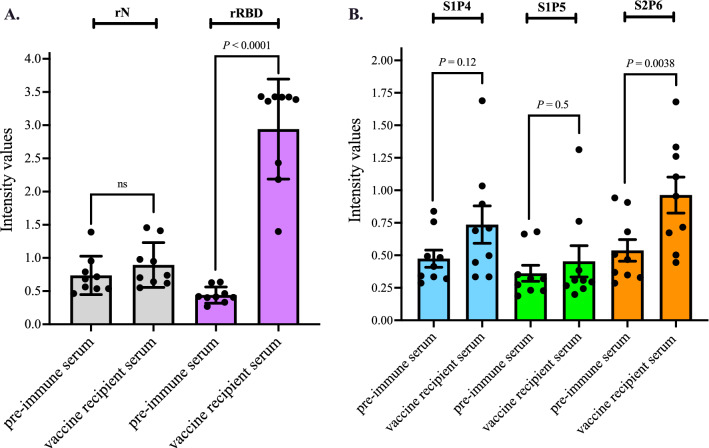
Immune reactivity of comirnaty vaccine recipient sera against the synthetic S1P4 and S2P6 peptides. Serum samples from comirnaty vaccine recipients (n = 9) (Table S4) were collected few weeks after the receipt of the second vaccine dose. The pre-immune serum of each individual was collected prior vaccination. In (**A**), serum samples at dilution 1:100 have been tested for SARS-CoV-2 N and S antibodies by indirect ELISA using soluble rN and rRBD proteins for antigen-based antibody capture. The intensity values of serum samples were measured at O.D. 450 nm. Paired *t* tests were performed between pre-immune serum and vaccine recipient serum (ns: non-statistically significant, *p* > 0.05). In (**B**), the serum samples were assayed on the synthetic S2P4 and S2P6 peptides (200 ng.ml^−1^) through peptide-based ELISA at serum dilution 1:50. The synthetic S1P5 peptide served as negative control serum. The intensity values of vaccine serum samples were measured at O.D. 450 nm. Paired *t* tests were performed between pre-immune serum and vaccine recipient serum.

We next evaluated the antigenic reactivity of the synthetic S1P4 and S2P6 peptides using a group of infection-naïve individuals who had been immunized with comirnaty vaccine in January–February 2021 (*n* = 9, median age: 50, female to male ratio: 0.9) (Table S4). The serum samples were collected few weeks after injection of a second dose of comirnaty vaccine. The pre-immune serum of each vaccinated subject was used as negative serum control. The comirnaty vaccine recipient sera (serum dilution 1:100) were first assessed for anti-RBD antibodies by indirect ELISA (Fig. 5A). All vaccinated individuals developed anti-RBD antibodies confirming that administration of comirnaty vaccine was effective in eliciting an antibody response against SARS-CoV-2 spike protein. The absence of anti-rN antibodies in comirnaty vaccine recipient sera confirmed that all nine subjects were infection-naïve individuals (Fig. 5A). Synthetic peptide-based ELISA was performed with the serum samples (dilution 1:50) from this group of comirnaty vaccine recipients (Fig. 5B). The peptide S1P5 served as negative peptide control. The synthetic S2P6 peptide but not the synthetic S1P4 peptide showed a significant antigenic reactivity with the comirnaty vaccine recipient sera as compared with pre-immune sera. The fact that comirnaty vaccine recipients had antibodies targeting the synthetic S2P6 peptide highlights for the ability of the stretch of residues 1138/1169 to be recognized as linear B-cell epitope in the recombinant spike protein expressed by comirnaty vaccine. Because the immune reactivity against the synthetic S1P4 peptide was not significantly different between the nine comirnaty vaccine recipient sera and their pre-immune sera, it is likely that individual differences exist in the development of an effective antibody response targeting the stretch of spike residues 616/644.

### Immune reactivity of COVID-19 patient sera with the synthetic S1P4 and S2P6 peptides

We evaluated antigenic reactivity of the synthetic S1P4 and S2P6 peptides with a group of COVID-19 immune subjects. We selected a panel of thirty COVID-19 donor sera (median age: 52.5 years, female to male ratio: 4) collected in November and December 2020 in France (Table S2). The COVID-19 serum donors developed mild COVID-19 symptoms without requirement for hospital admission. Symptomatic COVID-19 patients with progression to severe disease or admitted in intensive care units in hospital were not included in this study. Serological tests based on semi-quantitative SARS-CoV-2 specific antibody assays indicated that COVID-19 immune subjects developed anti-SARS-CoV-2 IgG and most of them also had specific IgM titers (Table S2). There were no significant differences in humoral immune reactivity among COVID-19 immune subjects related to women/men ratio or different age categories. Although durability of IgM and IgG can vary among COVID-19 patients, the IgM titers are consistent with serum samples collected within the first weeks following the acute infection.

**Figure 6:**
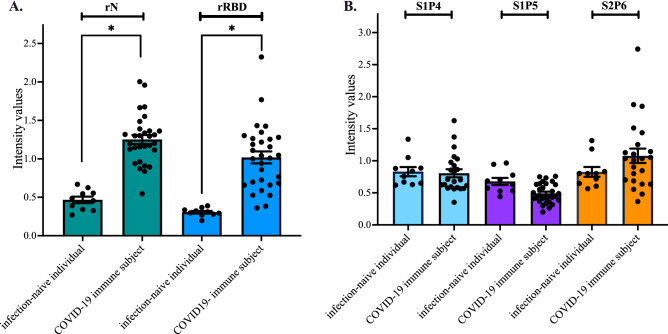
Antigenic reactivity of the synthetic peptides with human serum-specific SARS-CoV-2 antibody. Serum samples from COVID-19 recovered patients (*n* = 30) (Table S2) and ten infection- naïve individuals (*n* = 10) (Table S3) were tested for the antibody reactivity against the N and RBD proteins through indirect ELISA (**A**), or the S1P4, S1P5, and S2P6 peptides through synthetic peptide-based ELISA (**B**). The intensity values of serum samples were measured at O.D. 450 nm. In (**A**), the serum samples at dilution 1:100 were assayed for the detection of antibodies against SARS-CoV-2 N and spike proteins by indirect ELISA using recombinant rN and rRBD proteins (1 µg.ml^−1^) for antigen-based antibody capture. The intensity values of serum samples were measured at O.D. 450 nm. Unpaired *t* tests between infection-naïve individuals and COVID-19 immune subjects were performed and statistically significant comparisons are shown as * *p* < 10^–4^. In (**B**), The serum samples (*n* = 23) among the COVID-19 recovered patients who developed higher anti-RBD antibody titers were assayed for the detection of antibodies against the S1P4, S1P5, and S2P6 peptides (200 ng.ml^−1^) through synthetic peptide-based ELISA at serum dilution 1:50. The results are the mean (± SEM) of three replicates. The intensity values of serum samples were measured at O.D. 450 nm. Unpaired *t* tests between infection-naïve individual and COVID-19 immune subject were performed and differences between experimental points considered as non-statistically significant (*p* > 0.05) are not shown.

The immune reactivity of COVID-19 donor sera (Table S2) was first assessed for the presence of anti-spike antibodies by indirect ELISA using rRBD for antigen-based antibody capture (Fig. 6A). The rN protein was used as control viral antigen for natural SARS-CoV-2 infection. The serum samples collected before the emergence of COVID-19 in France were used as negative control sera (Table S3). There was a significant immune reactivity of COVID-19 donor sera against both rN and rRBD proteins. The serum samples (n = 23) among the COVID-19 recovered patients who developed higher anti-RBD antibody titers (serum dilution 1:100) were assayed through synthetic peptide-based ELISA (Fig. 6B). A lack of significant antigenic reactivity for S1P4 peptide with COVID-19 donor sera was observed (Fig. 6B). Serum samples from COVID-19 immune subjects showed no significant reactivity with the S2P6 peptide although a great variability in antibody response of relevant specificity suggesting that there might be several populations of responders to natural infection. Taken together, these results suggest that the stretches of spike residues 615/644 and 1138/1169 are not identified as immunodominant B-cell epitope peptides in the COVID-19 immune subjects who had experienced mild SARS-CoV-2 infection.

### Immunogenicity of synthetic S1P4 and S2P6 peptides in mice

To evaluate the in vivo immunogenicity of the two B-cell epitope peptides recognized by the comirnaty vaccine recipient sera, the synthetic S1P4 and S2P6 peptides were N-terminally coupled to Keyhole Limpet Hemocyanin (KLH) protein carrier. The protein-peptide conjugates were assessed with serum sample from a COVID-19 immune subject who received comirnaty vaccine. The KLH-S1P5 served as protein-peptide conjugate control. The antigenic reactivity of KLH-S1P4 and KLH-S2P6 conjugates was verified by indirect ELISA using a comirnaty vaccine recipient serum (Fig. S2A). The antigenic reactivity of protein-peptide conjugates was comparable to that observed with synthetic S1P4 and S2P6 peptides (Fig. S2B), confirming that both KLH-S1P4 and KLH-S2P6 are suitable for further experiments.

**Figure 7:**
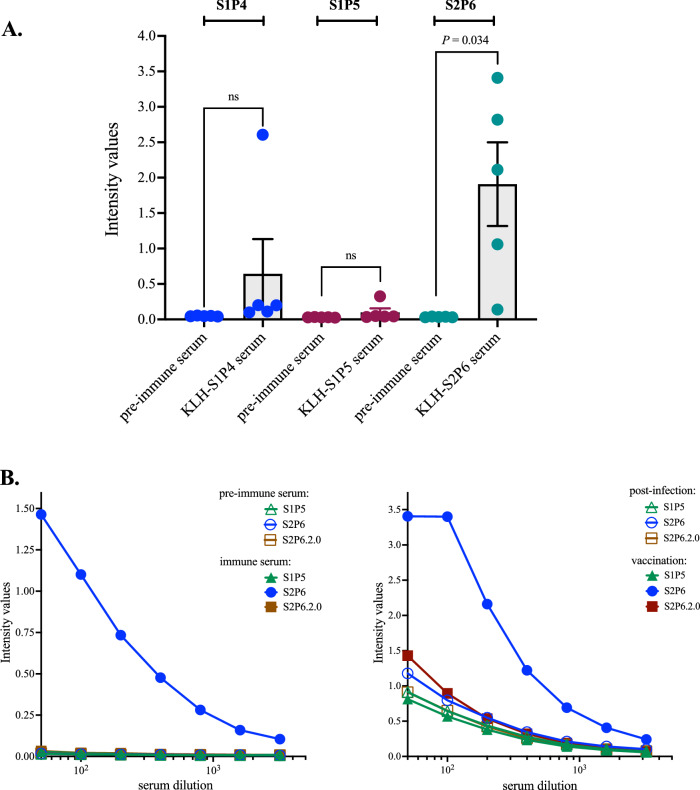
Immune reactivity of mouse antibodies raised against B-cell epitope peptides. In (**A**), serum samples from mice (*n* = 5) that received the KLH-peptide conjugates were assessed for peptide-reactive antibodies through peptide-based ELISA using the synthetic S1P4, S1P5, and S2P6 peptides (200 ng.mL^−1^) for antigen-based antibody capture. The pre-immune serum of each individual that received the KLH-peptide conjugates was tested. The intensity values of serum samples at dilution 1:50 were measured at O.D. 450 nm. Paired *t* tests between pre-immune serum and KLH-peptide serum were performed (ns: non-statistically significant, *p* > 0.05). The results are representative of two independent experiments. In (**B**), synthetic peptide-based ELISA using the S2P6 and S2P6.2.0 peptides (200 ng.mL^−1^) for peptide-based antibody capture. At the left, pre-immune and immune serum samples of an immunized mouse with KLH-S2P6 conjugates. At the right, serum samples from a COVID-19 immune subject collected after recovery (post-infection) and after injection of a single dose of comirnaty vaccine (vaccination). Synthetic S1P5 peptide served as negative peptide control. The intensity values of serum samples in a dose-curve response were measured at O.D. 450 nm.

To assess the immunogenicity of KLH-peptide conjugates in inbred laboratory mice, three groups of adult BALB/c mice were subcutaneously (s.c.) inoculated with 20–30 µg of KLH-S1P4, KLH-S1P5 or KLH-S2P6 conjugates in a prime-boost schedule. Immune sera were collected two weeks after the third immunization. The ability of the protein-peptide conjugates to elicit antibody response in mice was assessed by indirect ELISA using protein-peptide conjugates for antigen-mediated antibody capture. Immunization with KLH-S1P4 and KLH-S2P6 conjugates but not KLH-S1P5 conjugate elicited a strong antibody response against protein-peptide conjugates (Fig. S3). To evaluate the ability of the KLH-peptide conjugates to elicit antibody production of relevant specificity, individual mouse immune sera were tested on the synthetic S1P4, S1P5, and S2P6 peptides through a peptide-based ELISA (Fig. 7A). Mouse pre-immune serum served as control serum. Most of BALB/c mice (*n* = 5) that received KLH-S2P6 conjugates developed S2P6 peptide-reactive antibodies with a median O.D._450 nm_ value about 2.0 at serum dilution 1:50 (Fig. 7A). In contrast, the KLH-S1P4 conjugates were poorly immunogenic in BALB/c mice whereas KLH-S1P5 conjugates were inefficient to elicit a significant production of specific antibodies. The immunogenicity of the synthetic S2P6 peptide reinforces the notion that the linear B-cell epitope peptide which is delimited by the spike residues 1138/1169 in recombinant SARS-CoV-2 spike protein would be a feature of the Pfizer-BioNTech COVID-19 vaccine comirnaty.

A tridimensional structure prediction of the synthetic S2P6 peptide suggests that the amino-acids 10 to 21 (spike residues 1147/1157) could form α-helix (Fig. S4). The identified α-helix might act as potential binding site which enables the antibody recognition of synthetic S2P6 peptide. This prompted us to evaluate whether anti-S2P6 antibodies could target a truncated form of the synthetic S2P6 peptide. Consequently, we developed a 19-mer synthetic S2P6.2.0. peptide containing the amino-acids 10 to 28 of the B-cell epitope S2P6 peptide (Table 1). By peptide-based ELISA, we observed a lack of antigenic reactivity of synthetic S2P6.2.0 peptide with a mouse immune serum wherein the S2P6 peptide-reactive antibody concentration was high and also serum sample from COVID-19 immune subject who received a single dose of comirnaty vaccine after recovery (Fig. 7B). Thus, the motif ^1147^SFKEELDKYFKNHTSPDVD^1165^ is not sufficient for the antibody recognition. Given that binding capability of the linear B-cell epitope S2P6 peptide was completely lost when the nine N-terminal and four C-terminal amino-acids were deleted, it may be inferred that motifs ^1138^YDPLQPELD^1146^ and ^1162^LGDI^1165^ are potentially critical residues for epitope-antibody interaction.

## Discussion

The Pfizer/BioNTech COVID-19 vaccine comirnaty, a mRNA-based vaccine against SARS-CoV-2 infection, is highly effective at preventing serious illness and death among individuals of diverse ages. With the aim to identify specific antibody epitopes in comirnaty vaccine, a set of potential linear B-cell epitope peptides in the SARS-CoV-2 spike protein was assayed for its antigenic reactivity against serum samples from comirnaty vaccine recipients and COVID-19 recovered patients.

In the present study, we showed that administration of comirnaty vaccine to infection-naïve individuals elicits production of antibodies capable of binding to the synthetic S2P6 peptide and to a lesser extent the synthetic S1P4 peptide, targeting the SARS-CoV-2 spike residues 1138/1169 and 616/644, respectively. The spike residues 616/644 are located at the C-terminal domain 2 (CTD2) of S1 subunit upstream of the spike furin-cleavage site RRAR at the junction of S1 and S2 subunits whereas the spike residues 1138/1169 are positioned at the junction of the CD and the HR2 in the C-terminal region of S2 subunit (Fig. 1B). In contrast to what it has been observed with comirnaty-vaccinated individuals, serological analysis of COVID-19 patients revealed a lack of reactivity of the S1P4 and S2P6 peptides with antibodies raised against SARS-CoV-2. We showed that administration of comirnaty vaccine to a COVID-19 recovered patient elicits a production of antibodies capable of binding to synthetic S1P4 and S2P6 peptides. Thus, the comirnaty vaccine is effective to elicit an antibody response targeting the SARS-CoV-2 spike residues 1138/1169 and to a lesser extent, the residues 616/644, in COVID-19 immune subjects. The recombinant full-length SARS-CoV-2 spike protein expressed by a such COVID-19 mRNA vaccine is stabilized in a prefusion state due to amino-acid substitutions K986P and V987P^13,22,24^. One privileged hypothesis is that the immune recognition of spike residues 616/644 and 1138/1169 as linear B-cell epitope peptides would be an antigenic specificity of the pre-fusion stabilized spike protein. Whether the two B-cell epitope peptides representing the SARS-COV-2 spike residues 616/644 and 1138/1169 are a feature of comirnaty vaccine is an important issue that remains to be investigated. This could be achieved by analyzing a larger cohort of comirnaty-vaccinated individuals with or without prior SARS-CoV-2 infection^35^. It would also be of priority to evaluate whether the other commercially available COVID-19 mRNA vaccine mRNA-1273 licensed as spikevax by Moderna/NIAID also elicits an antibody response targeting the same stretches of spike residues 616/644 and 1138/1169^13,25^.

Phylogenetic analysis of SARS-CoV-2 variants indicated that the spike residues 616/644 and 1138/1169 are strictly conserved among identified VOCs including the Delta and Omicron variants. Also, the residues 1138/1169 mapped in the S2 subunit of spike protein are strictly conserved between SARS-CoV-2 and SARS-CoV. Taking into consideration with the singularity of the synthetic S1P4 and S2P6 peptides, it could be interesting to design peptide-based diagnosis assays to monitor antibody responses in comirnaty-vaccinated individuals without prior evidence of infection or with confirmed prior COVID-19 infection^36^. Indeed, the design of a such serological assay would be of interest to test for anti-spike antibody levels before administration of vaccine booster doses.

To our knowledge, it is not yet known whether the SARS-CoV-2 spike residues 616/644 and 1138/1169 contribute to the protective efficacy of Pfizer-BioNTech COVID-19 vaccine comirnaty^23,31–34,37,38^. There is some evidence that potent neutralization of SARS-CoV-2 requires both spike conformational changes and receptor blockade elicited by antibody binding^10,13–18,38^. The antibody recognition of the spike residues 616/644 into the C-terminal domain 2 of S1 subunit might have an impact on the efficiency of S1/S2 processing at the spike residue 685 by the cell surface protease TMPRSS2^15–20^. The antibody recognition of spike residues 1138/1169 might have an effect on fusion process between viral and host-cell membranes by trapping the prefusion state of spike protein timer^21,22^. Immunization of adult BALB/c mice with the synthetic S2P6 peptide conjugated to a protein carrier identified the SARS-CoV-2 spike residues 1138/1169 as immunogenic peptide. The SARS-CoV-2 spike protein was recognized by the S2P6 peptide-reactive antibodies suggesting that the synthetic S2P6 peptide could induce antibody response against SARS-CoV-2. Although truncation of the synthetic S2P6 peptide has not allowed to identify a minimal motif for antibody recognition, we assume that the N-terminal part of the B-cell epitope containing the spike residues 1138/1169 might play a key role in the epitope-antibody interaction. It is of priority to generate mouse hybridoma cell lines stably secreting monoclonal antibodies (mAbs) raised against the B-cell epitope peptide S2P6. Such anti-S2P6 mAbs will be assayed for their immune reactivity with spike protein trimer and neutralizing activity against live SARS-CoV-2.

Despite the lack of information on the protection conferred by the S2P6 peptide-reactive antibodies against SARS-CoV-2 infection, the data obtained with the B-cell epitope S2P6 peptide broadens our understanding on the efficacy of COVID-19 mRNA comirnaty vaccine. Finally, our study raises the question on the medical applicability of a vaccine formulation based on linear B-cell epitope peptides, e.g., the S2P6 peptide containing the residues 1138/1169 located in the C-terminal region of S2 subunit from SARS-CoV-2 spike protein^37–39^.

## Methods

### Antibody epitope mapping

The IEDB (for Immune Epitope Database, https://www.ied.org) as B-cell epitope-prediction tool was applied to the B-cell epitope candidates in the SARS-CoV-2 spike protein (UniprotKB-P0DTC2 (SPIKE_SRAS2)). The BepiPred-2.0 algorithm was used to predict antibody epitopes using 0.50 as the cut-off parameter.

### Expression of recombinant SARS-CoV-2 proteins in mammalian cells

Mammalian codon-optimized genes coding either for the SARS-CoV-2 N protein or the RBD domain of spike protein (residues 328/583) (UniProtKB-P0DTC9 (NCAP_SARS2)) were established using *Cricetulus griseus* codon usage as reference^40^. The sequence encoding the mouse Ig gamma-2A (IgG2A) chain C region sequence (UniprotKB-P01863 (GCAA_MOUSE) was fused in frame to the carboxy-terminus of N and RBD proteins with a spacer serine-glycine spacer. The protein sequence is preceded by a Secrecon signal peptide. The synthesis of gene sequences and their cloning into *Kpn*-I and *Xho*-I restriction sites of the pcDNA3.1 vector plasmid to generate pcDNA3/N-IgG2A and pcDNA3/RBD-IgG2A were performed by Genecust (Boynes, France). The plasmid sequences were verified by Sanger method (Genecust). Chinese Hamster Ovary CHO cells were transiently transfected with pcDNA3/N-IgG2A and pcDNA3/RBD-IgG2A using Lipofectamine 3,000 (Thermo Fisher Scientific, les Ulis, France). After 72 h of transfection, cell supernatants were harvested and clarified cell culture supernatant (C3S) fractions were obtained by centrifugation at 1000×*g* for 10 min at room temperature. Secreted N-IgG2a (rN) et RBD-IgG2a (rRBD) proteins in C3S fractions were detected by immunoblot assay using goat anti-mouse IgG heavy chain HRP antibody (Abcam, Cambridge, UK). The amounts of soluble rN and rRBD proteins in C3S fractions were estimated at 1.5 and 0.8 µg.mL^−1^, respectively, by direct ELISA test using goat anti-mouse IgG heavy chain HRP antibody. Standard for ELISA calibration was done using a serial dilution of mouse IgG at 1 mg.mL^−1^ as coating antigen (Vectors, Clinisciences, France).

### Synthetic SARS-CoV-2 spike peptides

All synthetic peptides used in this study were chemically synthesized by Genecust (Boynes, France). Peptides were dissolved in DMSO at concentration of 10 mg.mL^−1^ and then diluted in sterile H_2_O at the final concentration 1 mg.mL^−1^. The stock peptide solutions were stored at − 80 °C. Working peptide solutions at the final concentration 0.2 mg.mL^−1^ in sterile H_2_O were stored at − 80 °C.

### ELISA methods

For indirect ELISA assay using recombinant soluble rN and rRBD proteins as antibody capture antigens, a 96-well plate was coated with 0.1 ml of CS3 fractions at final concentration of 0.15 µg.mL^−1^ of rN or 80 ng.mL^−1^ of rRBD in PBS at 4 °C overnight. For peptide-based ELISA, a 96-well plate was coated with 0.1 ml of peptide at final concentration of 0.2 µg.mL^−1^ in PBS at 4 °C overnight. For indirect ELISA, a 96-well plate was coated with 0.1 ml of KLH-peptide at final concentration of 1 µg.mL^−1^ in PBS at 4 °C overnight. At the end of incubation period, the solution was discarded, the wells were washed with PBS supplemented with 0.01% Tween-20 (PBST) and then incubated with a commercial ELISA blocking agent (EBA) at room temperature (RT) for 1 h. After washing with PBST, the wells were incubated with human serum sample at final dilution 1:50 in EBA or mouse serum sample at final dilution 1:100 at 37 °C for 2 h. After washing with PBST, the wells were incubated with goat anti-human IgG-HRP or goat anti-mouse IgG-HRP at final dilution 1:2000 in EBA at room temperature for 1 h. After washing with PBST and PBS, the wells were incubated with TMB substrate solution at RT for 3 min and the reaction was stopped with acidic stopping solution. Absorbance was measured using microplate reader at 450 nm.

### Commercial serum samples from COVID-19 immune subjects

Serum samples were collected from de-identified COVID-19 immune subjects (*n* = 30 total) in 2020 (Table S2) and infection-naïve individuals (donation in 2019; *n* = 10 total) (Table S3) in France. All serum samples were purchased from Cerba Xpert (Saint-Ouen l’Aumône, France).

### Serum samples from comirnaty vaccine recipients

Blood samples were collected from de-identified healthy comirnaty vaccine recipient adults (*n* = 9) by professional medical caregivers in the Centre Hospitalier Universitaire de La Reunion (CHU de La Reunion, France) (Table S4). Ethical approval for this study was obtained from Research Ethics Committee “*Comité de Protection des Personnes, Nord Ouest IV de Lille, France* (approval number EudraCT/ID-RCB 2020-A01253-36)”. Written informed consent was obtained from all volunteers before. Age, gender, origin, date of vaccination and date of specimen collection were collected for all volunteers. Study individuals were fasting before venous puncture. Blood was sampled in EDTA tubes and plasma was stored at − 80 °C. All methods were carried out in accordance with relevant guidelines and regulations. Blood samples were collected in infection-naïve individual prior vaccination and few weeks after the receipt of the second dose of comirnaty vaccine.

In this study, two anonymized comirnaty vaccine recipients who have given their consent for the use of their serum samples were also assessed for the reactivity of their antibodies. An infection-naïve individual (Male, 59 years, France) who had been vaccinated with comirnaty vaccine in a two-dose regimen in April 2021. Serum samples were collected 0.5 and 2 months after the receipt of the second dose. A COVID-19 immune subject (Female, 62 years, France) who received the comirnaty vaccine three months after recovery. Serum samples were collected few weeks after COVID-19 recovery in March 2021 and then 0.5 month after the receipt of a single dose of comirnaty vaccine in June 2021.

### Mouse experiment and ethical statement

The Animal Ethics Committee of CYROI n°114 approved all the animal experiments with reference APAFIS#32,599-2021060109058958v2 (July 2021). All animal procedures were performed in accordance with the European Union legislation for the protection of animals used for scientific purposes (Directive 2010/63/EU). The study was conducted following the guidelines of the Office Laboratory of Animal Care (agreement n° 974 001 A) at the Cyclotron and Biomedical Research CYROI platform, Sainte-Clotilde, La Reunion, France and in accordance with the ARRIVE guidelines (https://arriveguidelines.org). Three groups of five 6-week-old female BALB/cJRj mice (Janvier Labs, France) were hosted in individually ventilated plastic cages (5 animals per cage) under 50–60% humidity and 22–25 °C temperature in a 12/12-h light dark cycle. Food and drinking were provided ad libitum. The adult BALB/cJRj mice (Janvier Labs, France) were s.c. inoculated with KLH-peptide conjugates in complete Freund’s adjuvant (Sigma, France). The protein-peptide conjugate samples in a final volume of 0.1 ml were distributed over two injection sites. Immunized mice were boosted with the same protein-peptide conjugate in incomplete Freund’s adjuvant at Days 7 and 21 after primary immunization (Day 0). Two weeks after the last immunization, retro-orbital blood sampling was performed in anesthetized mice. All animals were daily observed to detect any stress or suffering.

### Statistical analysis

Unpaired and pairedt  *t *tests were used to compare quantitative data. GraphPad Prism 9 was used for all statistical analysis.

## Supplementary Information

Supplementary Information.
