# Supplementary material for: The SARS-CoV-2 spike residues 616/644 and 1138/1169 delineate two antibody epitopes in COVID-19 mRNA COMIRNATY vaccine (Pfizer/BioNTech)
Source: Sci Rep. 2022 Apr 9;12:5999. doi: 10.1038/s41598-022-10057-7 (PMC8994064; doi:10.1038/s41598-022-10057-7)
Supplement: Supplementary file 1 — Supplementary Information. [file 41598_2022_10057_MOESM1_ESM.pdf]

Table S1. The B cell epitope candidates in the SARS-CoV-2 spike protein using BepiPred-2.0 algorithm

Peptide n°	Amino acid sequence	Position	Length
1	SQCVNLTTRTQLPPAYTNSFTRGVY	13-37	25
2	FSNVTWFHAIHVSGTNGTKRFDN	59-81	23
3	DPFLGVYYHKNNKSWME	138-154	17
4	MDLEGKQGNFKNL	177-189	13
5	KHTPINLVRDLPQGFS	206-221	16
6	TPGDSSSGWTA	250-260	11
7	KSFTVEKGIYQTSNFRVQP	304-322	19
8	FPNITNLCPFGEVFNATRFASVYAWNRKRISNCVA	329-363	35
9	YNSASFSTFKCYGVSPTKLNDLCFT	369-393	25
10	GDEVRQIAPGQTGKIADYNYKLP	404-426	23
11	NLDSKVGGNYNYLYRLFRKSNLKPFERDISTEIYQAGSTPCNGVEGFNCYFPLQSYGFQPTN	440-501	62
12	ELLHAPATVCGPKKSTNLVKN	516-536	21
13	NCTEVPVAIHADQLTPT	616-632	17
14	RVYSTGSNVFQ	634-644	11
15	VNNSYECDIPI	656-666	11
16	ASYQTQTNSPPARRSVASQ	672-690	19
17	YTMSLGAENSVAYSNN	695-710	16
18	KQIYKTPPIKDFGGF	786-800	15
19	PDPSKPSK	807-814	8
20	LADAGFIKQYGDCLG	828-842	15
21	RNFYEPQIITTD	1107-1118	12
22	VNNTVYDPLQPELDSFKEELDKYFKNHTSPDVDLGDISGI	1133-1172	40
23	SCCKFDEDDSEPVLKG	1252-1267	16

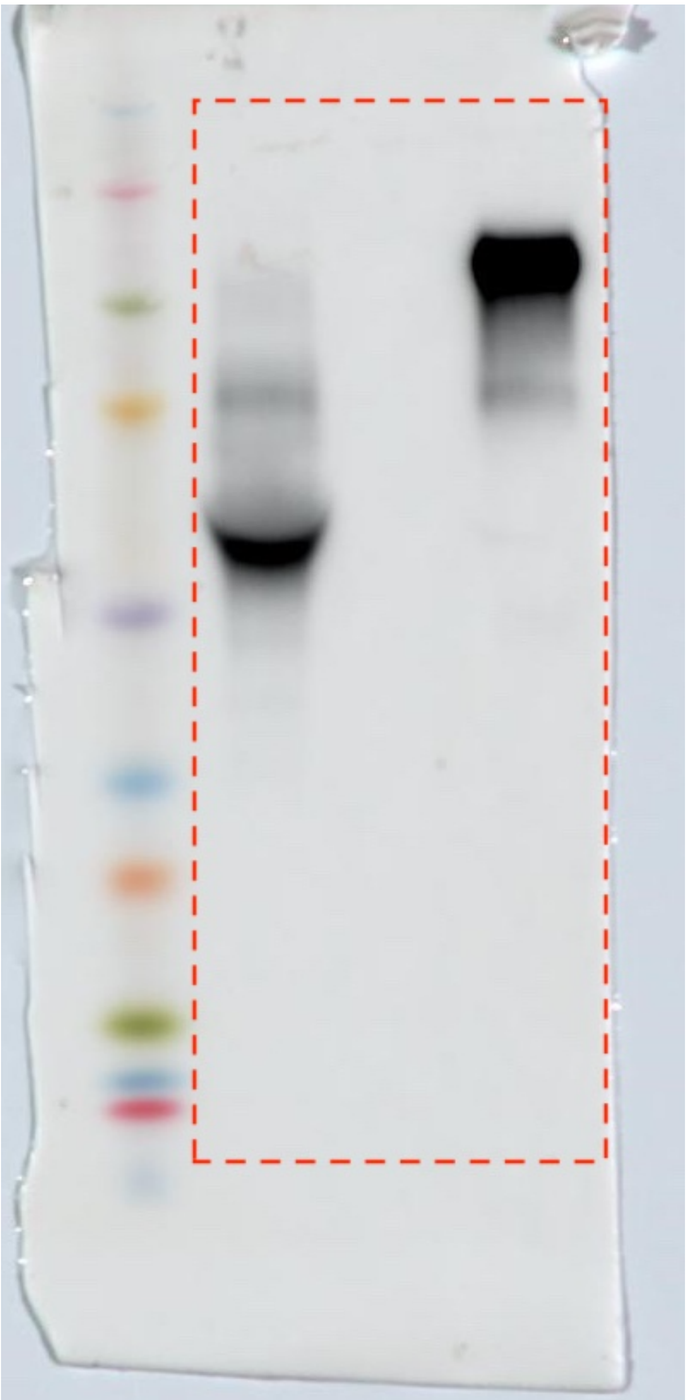

Figure S1. **Original and unprocessed immunoblot used in the Figure 2A.** The hatched red line depicts the portion of immunoblot gel shown in Fig. 2A.

Table S2. COVID-19 immune subject characteristics

donor n°	age[1]	gender	origin	date of specimen collection	IgM[2]	IgG[2]
1	13	F	France	11.20	+++	++
2	18	F	France	12.20	+	+++
3	23	M	France	11.20	++++	+++
4	23	M	France	11.20	++++	++
5	38	F	France	11.20	++++	+++
6	42	F	France	11.20	++++	+
7	45	F	France	12.20	++++	++
8	45	F	France	11.20	+	+++
9	46	F	France	11.20	+	+++
10	51	F	France	11.20	++	++
11	51	F	France	12.20	++	++
12	53	F	France	11.20	++++	++
13	59	M	France	11.20	+	+++
14	59	F	France	12.20	+++	+++
15	61	M	France	11.20	+++	+++
16	63	F	France	11.20	++	++++
17	64	F	France	11.20	+++	++
18	70	F	France	12.20	+	++++
19	71	F	France	12.20	++	++++
20	73	M	France	11.20	+	++++
21	77	F	France	11.20	+	++
22	79	F	France	12.20	+	++
23	80	F	France	12.20	++	+++
24	81	F	France	11.20	+	+++
25	85	F	France	11.20	++	++
26	85	F	France	11.20	++	++
27	87	F	France	11.20	+	+++
28	91	M	France	11.20	++	+++
29	91	F	France	11.20	++++	+++
30	97	F	France	11.20	+	++

[1.] years.

[2.] anti-SARS-CoV-2 antibodies fold increase of normal range as a score: + (1.5-2.0), ++ (2.0-4.0), +++ (4.0-6.0), ++++ (> 6.0).

<u>Table S3.</u> Infection-naïve individual characteristics

donor n°	age[1]	gender	origin	date of specimen collection
1	17	F	France	09.19
2	23	F	France	09.19
3	27	M	France	09.19
4	36	F	France	09.19
5	41	F	France	09.19
6	45	M	France	09.19
7	54	M	France	09.19
8	56	M	France	09.19
9	64	M	France	09.19
10	74	F	France	09.19

[1].years.

Table S4. COMIRNATY vaccine recipient characteristics.

donor n°	age[1]	gender	origin	date of vaccination	date of specimen collection
1	32	F	France[2]	03.21	03.21
2	33	M	France	02.21	03.21
3	39	M	France	01.21	02.21
4	44	F	France	02.21	02.21
5	50	F	France	02.21	03.21
6	55	M	France	02.21	03.21
7	56	F	France	02.21	03.21
8	58	M	France	02.21	02.21
9	62	M	France	02.21	03.21

1. years
2. La Reunion island (France)

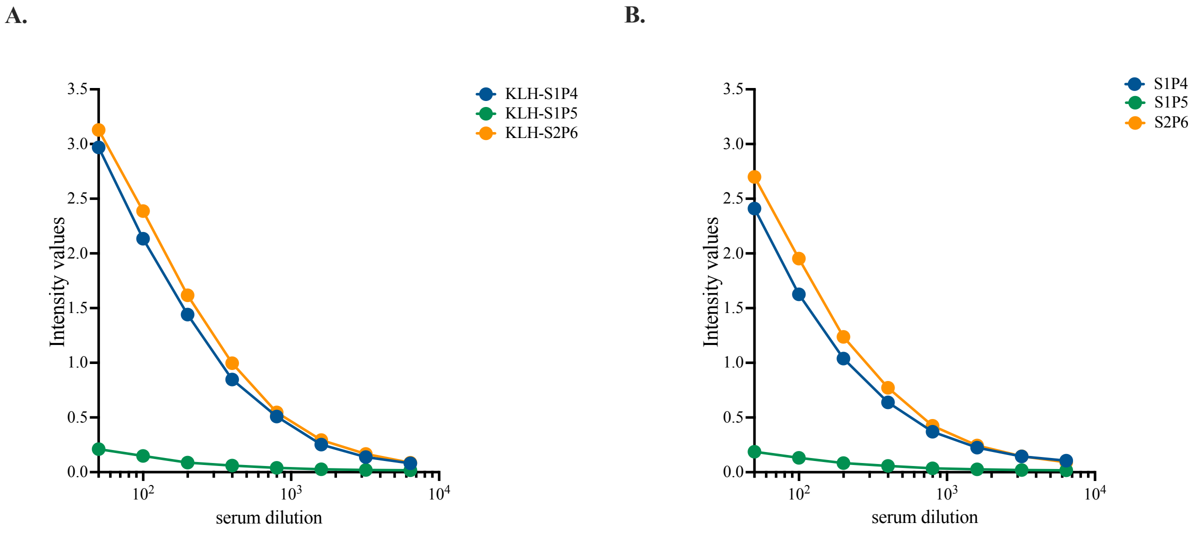

Figure S2. **Antigenic reactivity of protein-peptide conjugates**. Serum sample from a COVID-19 recovered patient who had been immunized with a single dose of COMIRNATY vaccine was collected 0.5 month after vaccine administration and serum dilutions were assayed on KLH-peptide conjugates through indirect ELISA (A) or synthetic peptides through peptide-based ELISA (B). The synthetic KLH-S1P5 conjugate and synthetic S1P5 peptide served as negative controls. The intensity values of serum samples were measured at O.D. 450 nm.

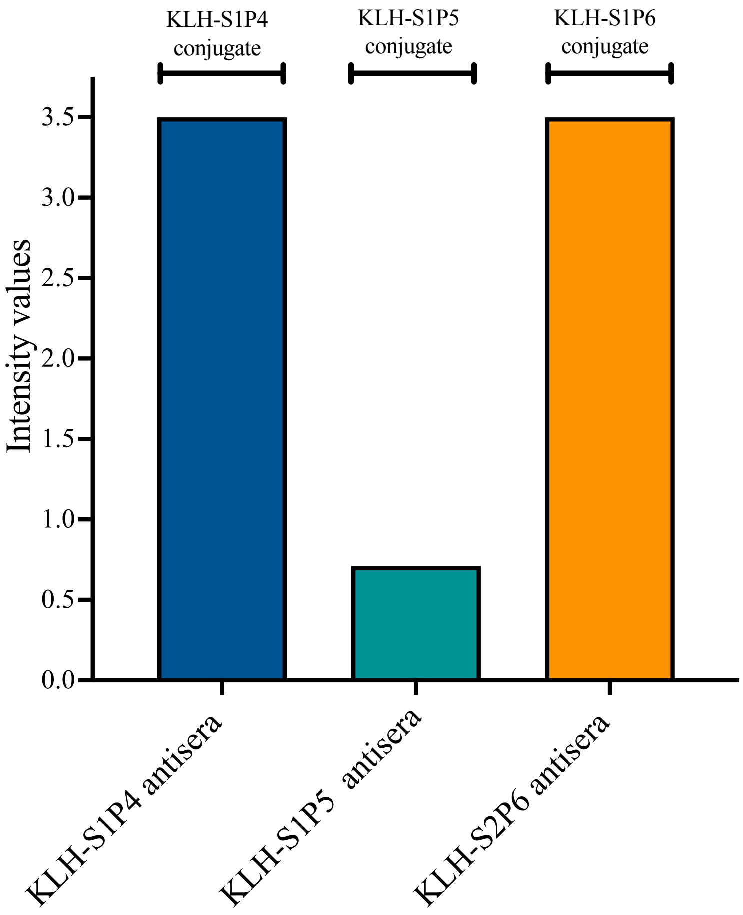

Fig. S3. **Immune reactivity of mouse antisera raised against protein-peptide conjugates.** Pools of serum samples from five mice that received KLH-peptide S1P4 (KLH-peptide S1P4 antisera), KLH-peptide S1P5 (KLH-peptide S1P5 antisera), or KLH-peptide S2P6 (KLH-peptide S2P6 antisera) in a prime-boost regimen were tested at serum dilution 1:100 for their immune reactivity in relation with protein-peptide conjugate (100 ng) by indirect ELISA. Intensity values or O.D. values at 450 nm.

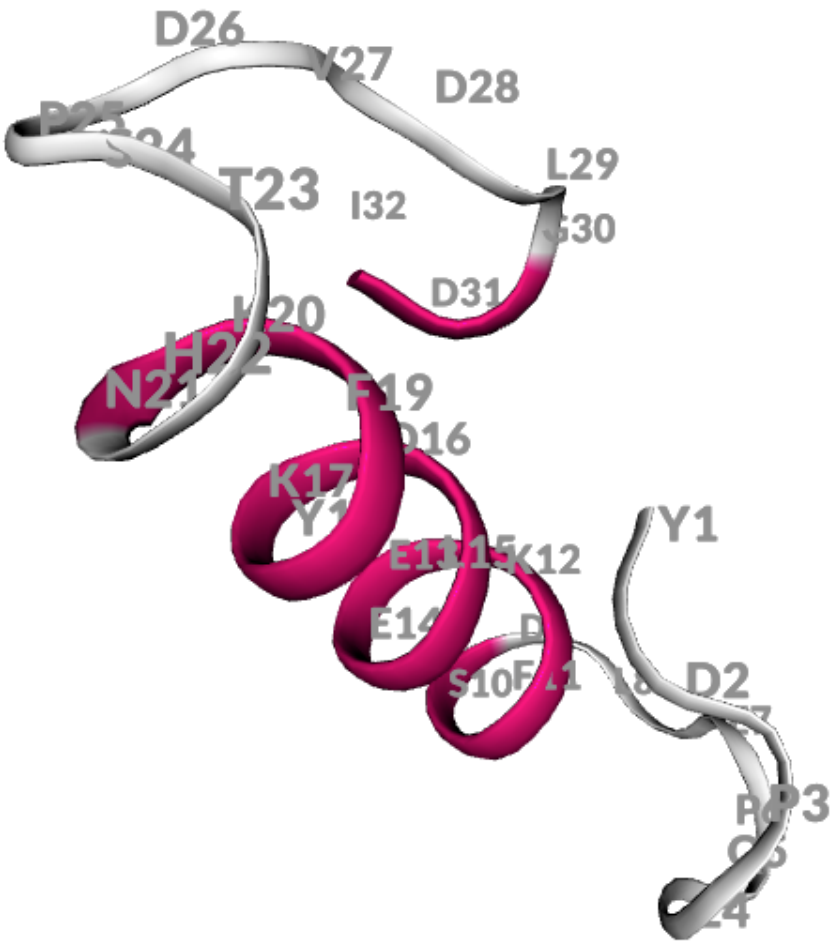

Fig.S4. **Tridimensional structure prediction of S2P6 peptide.** A 3D structure prediction of the B-cell epitope peptide S2P6 (residues Y1 to I32) was performed using PEP-FOLD3 protein recognition server (https://mobyle.rpbs.univ-paris-diderot.fr/cgi-bin/portal.py#forms::PEP-FOLD3) (34). The S2P6.2.0 peptide contains amino-acids S10 to D28.
